# From fistula to malignancy: rectal mucinous adenocarcinoma arising in a chronic perianal fistula, a rare diagnostic challenge

**DOI:** 10.1093/jscr/rjag1003

**Published:** 2026-10-31

**Authors:** Ahmed M AlMuhsin, Mohammed Tahtouh, Faisal Al Rashid, Zainab I Alruwaii, Abdulraheem Alshangiti, Fajer Bosaid

**Affiliations:** Department of General Surgery, Colorectal Surgery Section, King Fahad Specialist Hospital, Dammam, 32253, PO Box 31411, Kingdom of Saudi Arabia; Department of General Surgery, Colorectal Surgery Section, King Fahad Specialist Hospital, Dammam, 32253, PO Box 31411, Kingdom of Saudi Arabia; Department of General Surgery, Colorectal Surgery Section, King Fahad Specialist Hospital, Dammam, 32253, PO Box 31411, Kingdom of Saudi Arabia; Department of Pathology, King Fahad Specialist Hospital, Dammam, 32253, PO Box 31411, Kingdom of Saudi Arabia; Department of Adult Medical Oncology, King Fahad Specialist Hospital, Dammam, 32253, PO Box 31411, Kingdom of Saudi Arabia; Department of Medical Imaging, King Fahad Specialist Hospital, Dammam, 32253, PO Box 31411, Kingdom of Saudi Arabia

**Keywords:** mucinous adenocarcinoma, perianal fistula

## Abstract

Perianal fistula is a common surgical complaint that general surgeon frequently face in daily clinical practice. However, an adenocarcinoma arising from a fistula is an uncommon entity that has the propensity to mimic benign conditions leading to a delays in diagnosis and management. We present a case of a 42-year-old patient with long standing history of perianal fistula. Given the prolonged nature of his disease further investigations were warranted. While colonoscopy ruled out luminal disease, pelvic magnetic resonance imaging and deep tissue biopsies confirmed the diagnosis of invasive adenocarcinoma with focal mucinous component. The patient required neoadjuvant treatment followed by extra levator abdominoperineal excision and pelvic floor reconstruction. At 5-year follow up, the patient didn’t have local recurrence or distant metastasis.

## Introduction

Colorectal cancer is a major health concern with a rising incidence of early-onset disease worldwide. It is the most frequently diagnosed cancer among men in Saudi Arabia and the third prevalent in women [1]. Histopathologically, it encompasses several subtypes, with the mucinous adenocarcinoma subtype being characterized by the presence of extracellular mucin comprising more than 50% of the tumor volume.

The association between anal fistula and malignancy is not well established. Rarely, a perianal fistula undergoes a malignant transformation by virtue of ongoing inflammation and subsequently cellular metaplasia [2].

In 1934, Rosser and Skir reported the first case of fistula-associated malignancy and proposed diagnostic criteria for these cases including a history of anal fistula of 10 years or more, mucus secretion, induration and pain at the fistula site, an internal fistula opening in the anal canal or anal crypt, and the absence of a primary luminal tumor [2]. The postulated pathogenesis by Irving involves malignant changes in small duplications in the bowel, malignant changes in the fistula itself, malignant changes in the intramuscular glands, breakdown of a primary anal canal neoplasm, and the spread of cancer cells from a lesion higher in the lumen of the bowel implanted as a secondary deposit [3].

Given the rarity of this entity, there remains a paucity of clinical guidelines on the appropriate diagnostic modality and best evidence-based treatment strategies. Herein, we report a case of mucinous adenocarcinoma arising from a chronic perianal fistula managed successfully with neoadjuvant (NA) chemoradiation followed by surgery.

## Case report

A 42-year-old male with no known significant past medical history, presented with a 5-year history of perianal pain and discharge. His surgical history was unremarkable. Clinical examination revealed multiple external fistula openings, without active discharge. Examination of the groins revealed no lymphadenopathy. Digital rectal examination revealed no evidence of lesions or masses. Pelvic magnetic resonance imaging (MRI) revealed an extensive inflammatory pseudo mass in the subcutaneous fat in both side of the gluteal cleft with numerous sinuses and cystic changes. A small low left inter-sphincteric perianal fistula was noted as well (Figs 1 and 2). Colonoscopy was significant for mucosal hyperemia in the rectum and the cecum; for which multiple biopsies were obtained. The histopathological examination revealed an unusual crypt abscess with no other pathological changes. There were no discrete lesions identified in colonoscopy otherwise.

**Figure 1:**
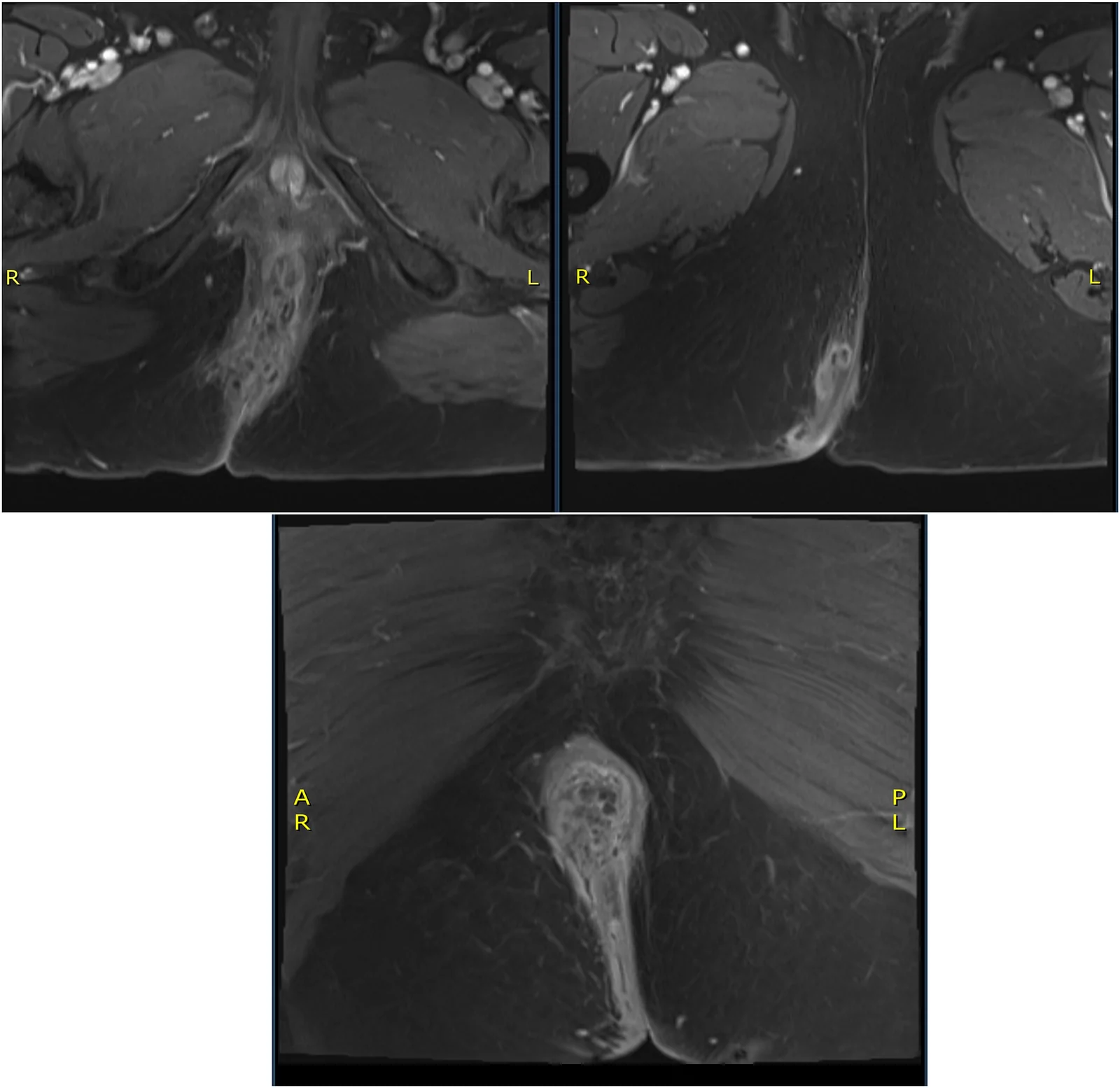
Axial and coronal post-contrast T1-weighted images demonstrate peripheral wall enhancement of the cystic foci with mild surrounding soft tissue enhancement.

**Figure 2:**
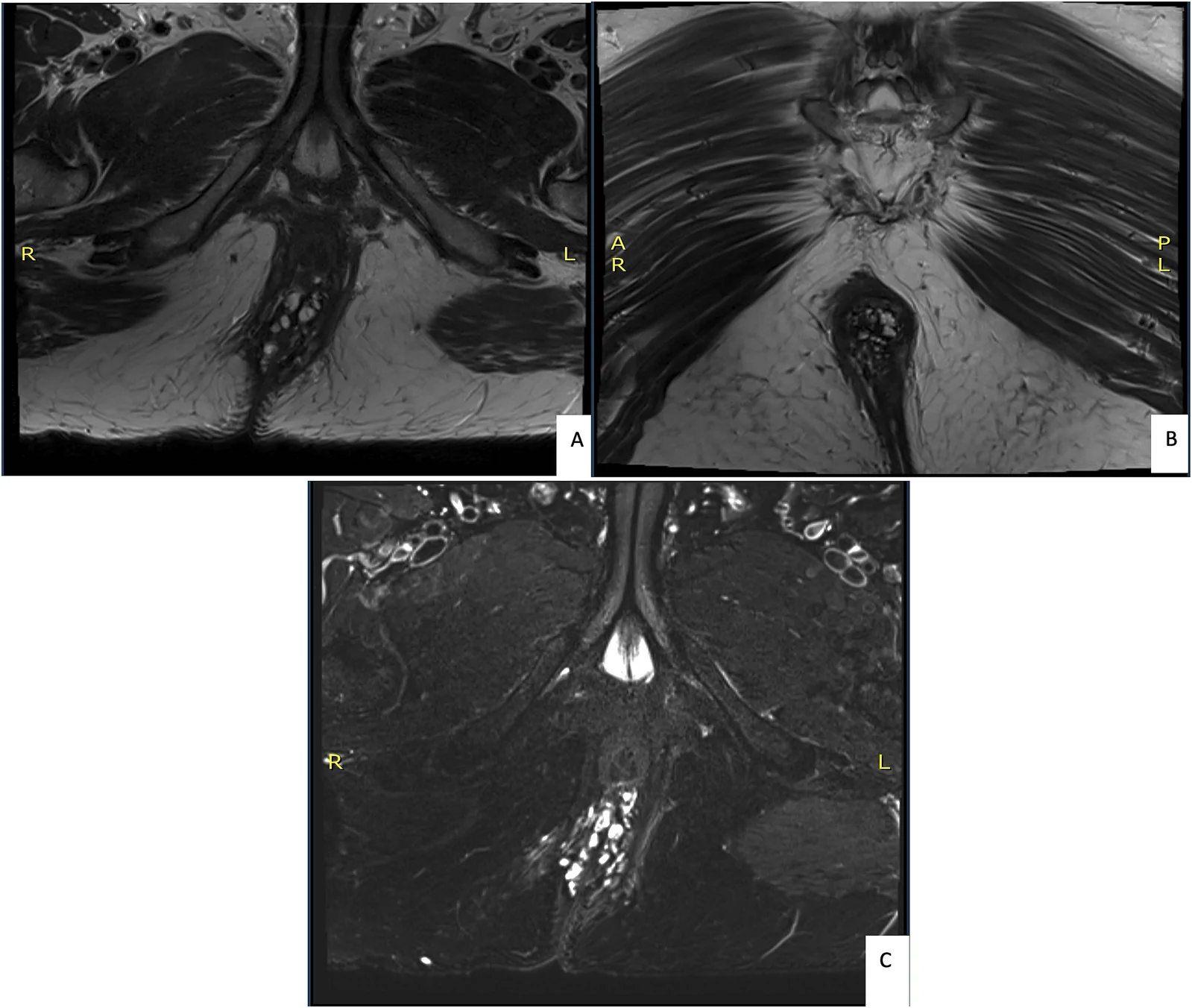
Axial (A) and coronal (B) T2-weighted images and axial T2 fat-suppressed images (C), demonstrate multiple intersphincteric perianal fistulous tracts at the 5, 6, and 7 o’clock positions. The tracts communicate caudally with innumerable grape-like T2 hyperintense cystic foci extending into the perineum, which are surrounded by a low T2 signal intensity soft tissue.

The patient subsequently underwent examination under anesthesia (EUA), at which biopsies were obtained from the fistula tract through curette and a draining seton was placed. Histopathology revealed an infiltrating well-differentiated adenocarcinoma, with focal mucinous component, arising in a background of skin with acanthosis, parakeratosis and marked dermal inflammation. Immunohistochemical stains showed the tumor cells were positive for Ck7, Ck20, and CDX2 (Fig. 3). Staging computed tomography of the chest, abdomen and pelvis showed a mildly enlarged inguinal lymph nodes bilaterally and an enlarged left external iliac lymph node measuring 1.1 and 1 cm in the short axis respectively. No evidence of distant metastasis was identified.

**Figure 3:**
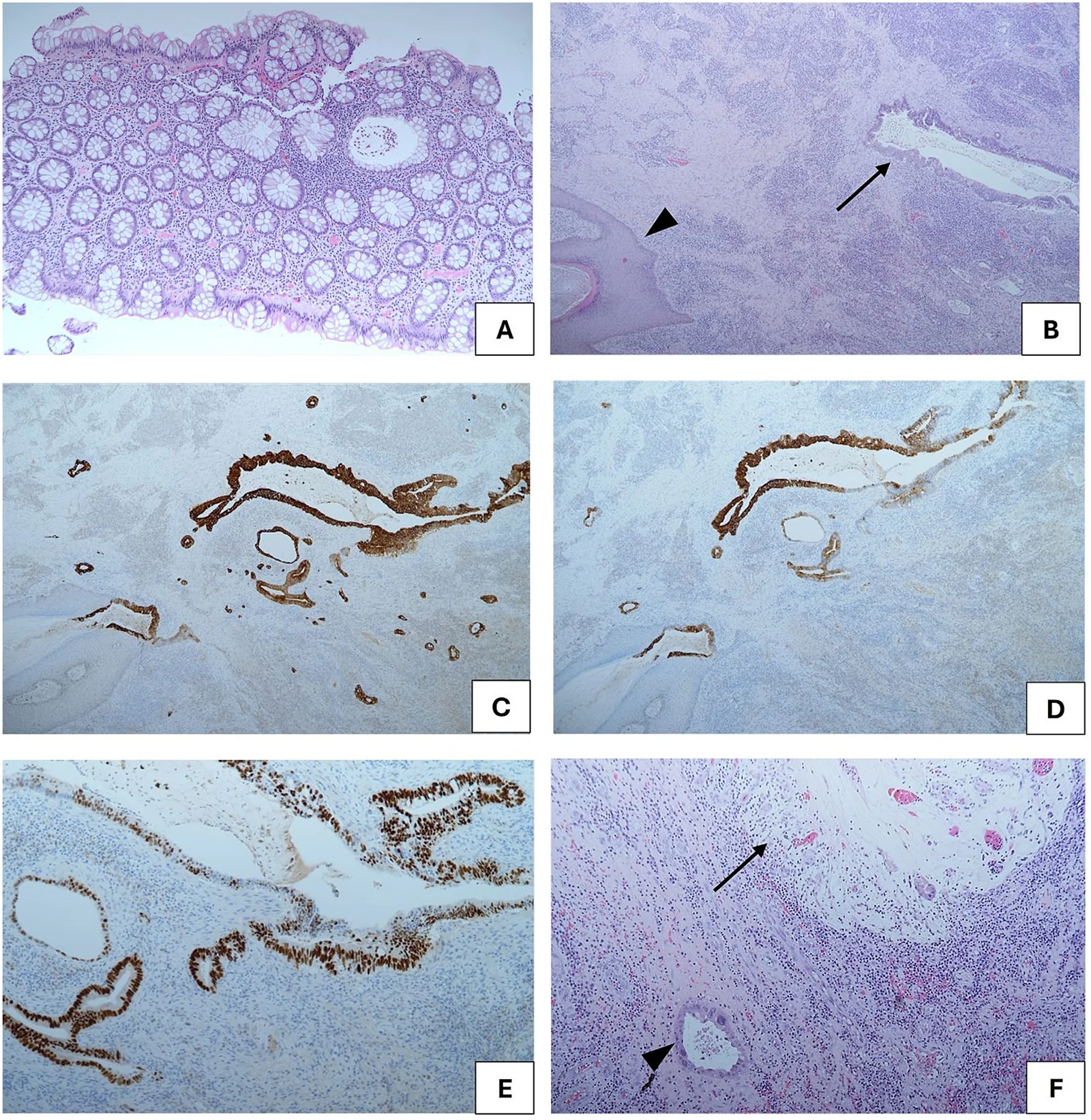
Histopathologic and immunohistochemical findings. (A) Initial rectal biopsy showing colonic mucosa with focal active inflammation and a crypt abscess, without evidence of dysplasia or malignancy (H&E, ×100). (B) Biopsy from the perianal fistula tract demonstrating inflamed skin (arrowhead) infiltrated by malignant glandular structures (arrow) (H&E, ×40). Immunohistochemical studies show the neoplastic glands are positive for pancytokeratin (C), CK20 (D), and CDX2 (E), supporting intestinal differentiation. (F) Higher-power photomicrograph demonstrating extracellular mucin pools (arrow), infiltrating malignant glands (arrowhead), and a background of chronic inflammation (H&E, ×100).

Following deliberation of the case by the multidisciplinary colorectal tumor board, the consensus was to initiate concurrent chemoradiotherapy. The patient completed 25 fractions of radiotherapy. Restaging pelvic MRI performed 8 weeks post radiation demonstrated a moderate response to treatment. Subsequently, the patient underwent an extralevator abdominoperineal excision and vertical rectus abdominis myocutaneous flap reconstruction 11 weeks post treatment. His postoperative course was complicated by perineal wound dehiscence which was managed with surgical wound debridement and negative pressure wound therapy, ultimately healing by secondary intention. Final pathological assessment of the specimen revealed a moderately differentiated adenocarcinoma, that invades the perineal skin, localized primarily around the fistula tract with no rectal involvement. No lymphovascular or perineural invasion were identified. Fourteen retrieved lymph nodes examined were negative and all margins were clear with more than 2 cm margin. The final pathological staging was ypT3N0. The patient didn’t require adjuvant chemotherapy. The patient maintained a regular follow up in the colorectal surgery clinic and was subject to regular investigations in accordance with colorectal cancer surveillance protocols. At 5 years follow up, there was no evidence of local recurrence or distant metastasis.

## Discussion

Perianal fistula is one of the most common presentations encountered in the colorectal and general surgery clinic. However, an adenocarcinoma of perianal fistula is a rare entity accounting for 0.3% of all fistulas [4]. A high index of suspicion is required to guide and establish the diagnosis, mainly in patients presenting with a long standing perianal fistula such as described in our report above, presence of mucinous secretion from the fistula opening, and absence of other pathology [4]. Nevertheless, there have been reports of an adenocarcinoma arising from perianal fistula within a much shorter period including durations as short as 3 months [5]. Moreover, malignancy arising within Crohn’s disease associated fistula has been reported, with an incidence of ~0.7% in a series of more than 1000 patients followed over a 14-year period [6].

The pathogenesis behind this entity remains unclear. It is hypothesized that malignancy occurs as a result of tumor implantation from a primary rectal cancer or as a result of local disease progression and less commonly due to prolonged inflammation within the fistula tract and subsequent cellular metaplasia [5]. Inflammatory cells, and various cytokines can act as tumor promoters by virtue of inducing oncogenic mutations that affect cellular proliferation and angiogenesis. Furthermore, free radicals can lead to oxidative damage and nitration of the DNA bases thereby increasing the risk of mutation [7]. The evolution of epithelial to mesenchymal transition (EMT) has been studied in the pathogenesis of Crohn’s disease associated fistula. Although EMT has been implicated in colorectal cancer development, it has not been studied in the setting of fistula related mucinous adenocarcinoma and the molecular links remain unclear [8].

The primary role of colonoscopy is to evaluate for an underlying primary colorectal cancer; thus, it represents an important first step in patient evaluation, even though it has a limited role in establishing the diagnosis of fistula associated malignancy. On the other hand, MRI provides critical imaging features useful in the diagnosis. Hyperintense signal on T2-weighted imaging can represent mucin pooling with heterogeneity and grape-like appearance differentiating it from pus. Additionally, the presence of mucin without a thick capsule can reflect the invasiveness of the disease as malignant cells would have invaded the perianal tissue before fibrosis occurred [9, 10]. Diffusion-weighted imaging (DWI) usually shows increased signal intensity, and the apparent diffusion coefficient can be used as a quantitative biomarker to differentiate mucinous from other histopathological subtypes. A less restricted diffusion in DWI usually represents mucin rather than pus [9, 10]. Ultimately, tissue biopsy will be required to establish a definitive diagnosis. Deep tissue biopsy and material debrided at the time of EUA will provide the pathologist with sufficient tissue to reach a histological diagnosis and avoid misinterpreting hyperplasia of the epithelial lining within the fistula tract with other benign entities [11].

There is no clear consensus on the best evidence-based management strategies for carcinoma arising from within fistula tracts. Many reports in the literature demonstrate successful management with surgery, or chemoradiation followed by surgery. Abdominoperineal resection (APR) was the mainstay of treatment in most of the reported cases. Kato *et al.* reported management with radiation followed by local excision with no signs of local recurrence at 21 months [12]. In 2003, Papagikos *et al.* reviewed 16 patients diagnosed with anal adenocarcinoma and found those with epidermoid histological features had a higher rate of local pelvic failure and distant metastasis when all have been treated with NA chemoradiation. Hence, they recommended managing these patient with NA chemoradiation followed by radical resection [13]. Conversely, Klas *et al.* reviewed 192 patients with anal carcinoma and argued surgery alone can achieve favorable outcome and the use of NA chemoradiation should be reserved for selected patients only [14]. Ong *et al.* reported four cases of mucinous adenocarcinoma arising from perianal fistula, three of whom were managed by APR alone and all had no local recurrence at 13, 40, and 39 months respectively [15]. More recently, Dahmeni *et al.* reported a complete pathological response following total NA treatment [16].

## Conclusion

Mucinous adenocarcinoma arising in the context of perianal fistula is rare, often leading to a delay in diagnosis. The surgeon should be vigilant when assessing a patient with chronic fistula, especially when associated with mucinous discharge. Colonoscopy and MRI play an important role in establishing the diagnosis. Although tissue biopsy carries a risk of a false negative result, it is imperative in assessing the invasive nature of the disease.

## References

[1] Aljabri AA, Alharbi AG. Colorectal cancer screening in Saudi Arabia: current challenges and future directions. Saudi Med J 2026;47:10–20. 10.15537/smj.2026.47.1.20250597 PMC12862581 41628970

[2] Rosser C . The relation of fistula-in-ano to cancer of the anal canal. Trans Am Proc Soc 1934;35:65–71.

[3] Parnes IH . An interesting case of cancer of the sigmoid with concomitant cancer in an anal fistula. Mt Sinai J Med 1976;43:476–9. 1085913

[4] Ishiyama S, Inoue S, Kobayashi K et al. Implantation of rectal cancer in an anal fistula: report of a case. Surg Today 2006;36:747–9. 10.1007/s00595-006-3236-3 16865523

[5] Yamada K, Miyakura Y, Koinuma K et al. Primary and secondary adenocarcinomas associated with anal fistulae. Surg Today 2014;44:888–96. 10.1007/s00595-013-0629-y 23722283

[6] Ky A, Sohn N, Weinstein MA et al. Carcinoma arising in anorectal fistulas of Crohn’s disease. Dis Colon Rectum 1998;41:992–6. 10.1007/BF02237388 9715154

[7] Hussain SP, Hofseth LJ, Harris CC. Radical causes of cancer. Nat Rev Cancer 2003;3:276–85. 10.1038/nrc1046 12671666

[8] Scharl M, Frei P, Frei SM et al. Epithelial-to-mesenchymal transition in a fistula-associated anal adenocarcinoma in a patient with long-standing Crohn’s disease. Eur J Gastroenterol Hepatol 2014;26:114–8. 10.1097/MEG.0b013e32836371a2 24284372

[9] Ho CM, Tan CH, Ho BCS. Clinics in diagnostic imaging (143). Perianal mucinous adenocarcinoma arising from chronic fistula-in-ano. Singapore Med J 2012;53:843–8. quiz p. 849. 23268160

[10] Hama Y, Makita K, Yamana T et al. Mucinous adenocarcinoma arising from fistula in ano: MRI findings. AJR Am J Roentgenol 2006;187:517–21. 10.2214/AJR.05.0011 16861558

[11] Onerheim RM . A case of perianal mucinous adenocarcinoma arising in a fistula-in-ano. A clue to the early pathologic diagnosis. Am J Clin Pathol 1988;89:809–12. 10.1093/ajcp/89.6.809 2835897

[12] Kato R, Miyamoto Y, Sawayama H et al. Relapse of rectal cancer in an anal fistula: a rare case. In Vivo 2021;35:2937–40. 10.21873/invivo.12585 PMC8408709 34410990

[13] Papagikos M, Crane CH, Skibber J et al. Chemoradiation for adenocarcinoma of the anus. Int J Radiat Oncol Biol Phys 2003;55:669–78. 10.1016/S0360-3016(02)04118-4 12573754

[14] Klas JV, Rothenberger DA, Wong WD et al. Malignant tumors of the anal canal: the spectrum of disease, treatment, and outcomes. Cancer 1999;85:1686–93. 10.1002/(SICI)1097-0142(19990415)85:8<1686::AID-CNCR7>3.0.CO;2-7 10223561

[15] Ong J, Jit-Fong L, Ming-Hian K et al. Perianal mucinous adenocarcinoma arising from chronic anorectal fistulae: a review from a single institution. Tech Coloproctol 2007;11:34–8. 10.1007/s10151-007-0322-5 17357864

[16] Dahmeni W, Souilem E, Hassen H et al. Complete pathological response to neoadjuvant chemoradiotherapy in Crohn’s disease–associated fistula adenocarcinoma: a case report. Front Med (Lausanne) 2026;13:1746760. 10.3389/fmed.2026.1746760 PMC13079112 41994432

